# Amelioration of Prallethrin-Induced Oxidative Stress and Hepatotoxicity in Rat by the Administration of *Origanum majorana* Essential Oil

**DOI:** 10.1155/2013/859085

**Published:** 2013-12-05

**Authors:** Abdel-Tawab H. Mossa, Amel A. Refaie, Amal Ramadan, Jalloul Bouajila

**Affiliations:** ^1^Environmental Toxicology Research Unit (ETRU), Pesticide Chemistry Department, National Research Centre (NRC), Tahrir Street, Dokki, Giza, Egypt; ^2^Department of Biochemistry, National Research Centre (NRC), Tahrir Street, Dokki, Giza, Egypt; ^3^Université de Toulouse, Faculté de Pharmacie de Toulouse, Université Paul-Sabatier, Laboratoire des IMRCP-UMR CNRS-UPS 5623, Cedex 9, 31062 Toulouse, France

## Abstract

This study was carried out to evaluate the adverse effects of exposure to prallethrin on oxidant/antioxidant status and liver dysfunction biomarkers and the protective role of *Origanum majorana* essential oil (EO) in rat. Male rats were divided into 4 groups: (i) received only olive oil (ii) treated with 64.0 mg/kg body weight prallethrin (1/10 LD_50_) in olive oil via oral route daily for 28 days, (iii) treated with 64.0 mg/kg body weight prallethrin (1/10 LD_50_) and EO (160 **μ**L/kg b.wt.) in olive oil and (iv) received EO (160 **μ**L/kg b.wt.) in olive oil via oral route twice daily for 28 days. Prallethrin treatment caused decrease in body weight gain and increase in relative liver weight. There was a significant increase in the activity of serum marker enzymes, aspartate transaminase, alanine transaminase, and alkaline phosphatase. It caused increase in thiobarbituric acid reactive substances and reduction in the activities of superoxide dismutase, catalase, and glutathione-S-transferase in liver. Consistent histological changes were found in the liver of prallethrin treatment. EO showed significant protection with the depletion of serum marker enzymes and replenishment of antioxidant status and brought all the values to near normal, indicating the protective effect of EO. We can conclude that prallethrin caused oxidative damage and liver injury in male rat and co-administration of EO attenuated the toxic effect of prallethrin. These results demonstrate that administration of EO may be useful, easy, and economical to protect human against pyrethroids toxic effects.

## 1. Introduction 

Synthetic pyrethroids are the newest major class of broad-spectrum organic insecticides used in agricultural, domestic, and veterinary applications. They are widely applied in view of the fact that they have shown to possess a high insecticidal activity as well as a broad spectrum of high initial toxic action on several types of pests [1]. Although they were not used commercially until 1980, pyrethroid insecticides accounted for more than 25% of the world market [2, 3]. Therefore, the widespread use of pyrethroid insecticides consequently leads to the exposure of manufacturing workers, field applicators, the ecosystem, and finally the public to the possible toxic effects of pyrethroid insecticides.

Pyrethroid insecticides are acute neurotoxicants [4], modulate the function of voltage-gated sodium channels [5]; specifically, they alter the permeability of excited nerve cells to sodium ions and cause repetitive nerve impulses [4, 6]. They also have other neurobiologic actions, including effects on central *γ*-amino butyric acid, noradrenergic, dopaminergic, and cholinergic neurotransmission [7]. However, the toxicity of pyrethroid insecticides to mammals has received much attention in recent years because animals exposed to these insecticides showed changes in their physiological activities besides other pathological features [8, 9]. Due to lipophilic nature of pyrethroid insecticides [10], they easily cross biological membranes but accumulate in biological membranes leading to stimulate the production of reactive oxygen species (ROS) and result in oxidative damage in mammals [11, 12] and aquatic organisms [13]. Oxidative stress and resulting damage to essential cell components caused by oxygen-free radicals are generally considered a serious mechanism. Previous studies suggested that some effects directly related to pesticide toxicity could be due to changes in membrane fluidity [14–16], in lipid composition [17], and inhibition of enzyme activities [18–20].

Prallethrin is the most popular Type I synthetic pyrethroid that produces a rapid knockdown in household insect pests such as mosquitoes, houseflies, and cockroaches [21]. It has prevalent household presence in the form of mosquito repellant mats, coils, liquid vaporizers, and so forth and therefore there could be direct and indirect exposure in pets and humans through accidental continued contamination of food and water [22].

Currently, there is an increased demand for using medicinal plants in therapy, in both developing and developed countries due to growing recognition of natural products, the “back to nature” slogan, instead of using synthetic drugs which might have adverse effects.* Origanum majorana *L. (*O. majorana*) is a member of the mint family Lamiaceae. In folk medicine, marjoram is used for cramps, depression, dizziness, gastrointestinal disorders, migraine, nervous headaches, and paroxysmal coughs and as a diuretic [23]. It contains phenolic terpenoids (thymol, carvacrol), flavonoids (diosmetin, luteolin, and apigenin), tannins, hydroquinone, phenolic glycosides (arbutin, methyl arbutin, vitexin, and orientinthymonin), triacontane, sitosterol, acids (oleanolic acid), and cis-sabinene hydrate [24, 25].

There are several reports on oils indicating that it results in alterations of pharmacologic responses to drugs [26]. In our previous study [27], *O. majorana* essential oil (EO) was analyzed by gas chromatography-mass spectrometry (GC-MS) and gas chromatography-flame ionization detector (GC-FID) and evaluated for free radical scavenging activities. GC-MS analysis revealed the presence of 4-terpineol (29.97%), *γ*-terpinene (15.40%), trans-sabinene hydrate (10.93%), *α*-terpinene (6.86%), and 3-cycolohexene-1-1 methanal,a,a4-trimethyl-,(S)-(CAS) (6.54%) as main constituents. It exhibited concentration-dependent inhibitory effects on DPPH^∙^, hydroxyl radical, hydrogen peroxide, reducing power, and lipid peroxidation [27].

At this time, a very little, unsatisfactory information is available in literature on oxidative stress and hepatotoxicity of prallethrin in mammals. In addition, the use of *O. majorana* EO to alleviate the oxidative damage and hepatotoxicity induced by pesticides has not been previously examined. Therefore, this study was interested first in evaluating the adverse effects of exposure to prallethrin on oxidant/antioxidant status and liver dysfunction biomarkers and second in the protective role of *O. majorana* EO against prallethrin-induced oxidative damage and hepatotoxicity in rat.

## 2. Materials and Methods

### 2.1. Materials

Prallethrin (96.2%) was obtained from Jiangsu Yangnong Chemical Co., Ltd, China. The assay kits used for biochemical measurements of catalase (EC 1.11.1.6), superoxide dismutase (EC 1.15.1.1), glutathione-S-transferase (EC 2.5.1.13), aspartate aminotransferases (EC 2.6.1.1.), alanine aminotransferases (EC 2.6.1.2), alkaline phosphatase (EC 3.1.3.1), and lipid peroxidation were purchased from Biodiagnostic Company, 29 Tahrir Street, Dokki, Giza, Egypt. Kit of protein was obtained from Stanbio Laboratory, Texas, USA. All other chemicals were of reagent grades and obtained from the local scientific distributors in Egypt.

### 2.2. Preparation of Essential Oil

*O. majorana *EO was obtained from leaves by hydrodistillation in a Clevenger apparatus. The distillation continued until no more condensing oil could be seen. The oil was permitted to stand undisturbed so that a good separation from water could be obtained. The essential oil was separated from the aqueous solution, dried over anhydrous sodium sulfate, transferred into an amber glass flask, and kept at a temperature of −20°C until used. *O. majorana* EO was identified by GC (THERMO TRACE 2000) equipped with a MS (FINNI-GAN SSQ 7000) GC-MS system (Central Laboratory of the National Research Centre, Cairo, Egypt) as described in our previous study [27].

### 2.3. Animals

Healthy male Wistar rats were obtained from Animal Breeding House of the National Research Centre (NRC), Dokki, Cairo, Egypt. Rats were housed in clean plastic cages with free access to food (standard pellet diet) and tap water *ad libitum*, under standardized housing conditions (12 h light/dark cycle, the temperature was 23 ± 2°C, and a minimum relative humidity of 44%) in the laboratory animal room. Animals received humane care according to the criteria outlined in the “Guide for the Care and Use of Laboratory Animals.” The Local Ethics Committee at the National Research Centre (NRC), Dokki, Cairo, Egypt, approved the experimental protocols and procedures. The rats attained a body weight range of 145–155 g before being used for this study.

### 2.4. Experimental Design

Dosages of prallethrin and *O. majorana* EO were freshly prepared in olive oil, given via oral route for 28 consecutive days, and adjusted weekly for body weight changes. The animals were acclimatized for a minimum of 7 days before treatment and randomly assigned into four groups of seven rats each. Rats in group one received olive oil and served as control. Group two received prallethrin at a dose 64.0 mg/kg b.wt. (1/10 LD_50_). Group three received prallethrin at a dose 64.0 mg/kg b.wt. and *O. majorana* EO at 160 *μ*L/kg b.wt. twice daily. Group four received *O. majorana* EO at a dose 160 *μ*L/kg b.wt. twice daily.

The selective dose of prallethrin is based on published LD_50_ (640 mg/kg b.wt.) [28], and dose of* O. majorana* EO (160 *μ*L/kg b.wt. twice daily) is based on El-Ashmawy et al. [29]. At the end of the administration, the animals were fasted for 12 hours and sacrificed by ether anesthesia with cervical dislocation on 29th day.

### 2.5. Body Weight and Samples Preparation

Body weights were recorded weekly during the experimental period (28 days). At the end of this period, blood samples were withdrawn from the animals under light ether anaesthesia by puncturing the retero-orbital venous plexus of the animals with a fine sterilized glass capillary. Blood samples were taken and left to clot in clean dry tubes and then centrifuged at 3000 rpm (600 g) for 10 minutes using Heraeus Labofuge 400R, Kendro Laboratory Products GmbH, Germany, to obtain the sera. The sera was then stored frozen at −20°C for the biochemical analysis (ALT, AST, and ALP). After blood collection, rats were then killed by decapitation, and livers were dissected out, cleaned, weighed. Small pieces of liver were cut and kept in 10% formalin solution for histological studies. Other portions of liver washed with saline solution, weighed, cut in small parts, homogenized in 10% (w/v) ice cold 100 mM phosphate buffer (pH 7.4) and centrifugation at 10,000 ×g for 15 minutes at 4°C, and then the supernatant was obtained and used for antioxidant enzyme measurements (CAT, SOD, and GST) and total protein.

### 2.6. Serum Liver Dysfunction Marker Enzymes

Serum aminotransferases (AST and ALT) and ALP were measured spectrophotometrically as described by Reitman and Frankel [30] and Young et al. [31], respectively, using Shimadzu UV-VIS Recording 2401 PC (Japan), performed according to the details given in the kit's instructions and were expressed in terms of U/L.

### 2.7. Liver Lipid Peroxidation and Antioxidant Enzymes

Antioxidant enzyme activities and lipid peroxidation were determined in liver homogenate. A centrifugation was carried out and thus, antioxidants were measured in the isolated cell fraction using a spectrophotometer Shimadzu UV-VIS Recording 2401 PC (Japan). It was performed according to the details given in the kit's instructions. The principals below of different methods are given for each concerned biochemical parameter.

### 2.8. Lipid Peroxidation

Lipid peroxidation was estimated by measuring thiobarbituric acid reactive substances (TBARS) and was expressed in terms of malondialdehyde (MDA) content by a colorimetric method according to Satoh [32]. The MDA values were expressed as nmoles of MDA/mL.

### 2.9. Antioxidant Enzymes

Superoxide dismutase activity was determined according to the method of Nishikimi et al. [33]. The method is based on the ability of SOD enzyme to inhibit the phenazine methosulphate-mediated reduction of nitroblue tetrazolium dye (NTB). Briefly, 0.05 mL sample was mixed with 1.0 mL buffer (pH 8.5), 0.1 mL nitroblue tetrazolium (NBT), and 0.1 mL NADH. The reaction was initiated by adding 0.01 mL phenazine methosulphate (PMs), and then increase in absorbance was read at 560 nm for five minutes. SOD activity was expressed in *μ*mol/mg protein.

Catalase activity was determined according to the method of Aebi [34]. The method is based on the decomposition of H_2_O_2_ by catalase. The sample containing catalase is incubated in the presence of a known concentration of H_2_O_2_. After incubation for exactly one minute, the reaction is quenched with sodium azide. The amount of H_2_O_2_ remaining in the reaction mixture is then determined by the oxidative coupling reaction of 4-aminophenazone (4-aminoantipyrene, AAP) and 3,5-dichloro-2-hydroxybenzenesulfonic acid (DHBS) in the presence of H_2_O_2_ and catalyzed by horseradish peroxidase (HRP). The resulting quinoneimine dye (N-(4-antipyrl)-3-chloro-5-sulfonate-p-benzoquinonemonoimine) is measured at 510 nm. The catalase activity was expressed in *μ*mol/mg protein.

Glutathione-s-transferase activity in the liver was assessed spectrophotometrically according to the method of Habig et al. [35]. The method was based on the conjugation of 1-chloro-2 4-dinitrobenzene (CDNB) with reduced Glutathione (GSH) in a reaction catalyzed by GST. Increase in absorbance was monitored for 3 min at 30 sec intervals at wavelength of 340 nm. Results were expressed as nmol/mg protein.

### 2.10. Protein Concentration

The total protein concentrations in homogenate were determined spectrophotometrically based on the colorimetric biuret method according to Gornall et al. [36].

### 2.11. Histological Study

After the end of the treatment period, rats were killed, and liver samples were dissected and fixed in 10% neutral formalin, dehydrated in ascending grades of alcohol, and imbedded in paraffin wax. Paraffin sections (5 *μ*m thick) were stained for routine histological study using haematoxylin and eosin (H&E). Two slides were prepared for each rat; each slide contains two sections. Ten field areas for each section were selected and examined for histopathological changes (x160) under light microscope. The liver fields were scored as follows: normal appearance (−), minimal cellular disruption in less than 1% of field area (+), mild cellular disruption of 1–30% of field area (++), moderate cellular disruption of 31–60% of field area (+++), severe cell disruption of 61–90% of field area (++++), and very severe cellular disruption of 91–100% of field area (++++). Previous investigators have performed such quantitative assessment of histopathological injury [37].

### 2.12. Statistical Analysis

The results were expressed as means ± S.E. All data were done with the Statistical Package for Social Sciences (SPSS 17.0 for windows). The results were analyzed using one way analysis of variance (ANOVA) followed by Duncan's test for comparison between different treatment groups. Statistical significance was set at *P* ≤ 0.05.

## 3. Results

### 3.1. Signs of Toxicity

No clinical signs of prallethrin poisoning were observed among rats of treated groups such as diarrhea, hair loss, nasal hemorrhage, and bloated abdomen. Moreover, death was not observed during experimental period (28 days).

### 3.2. Body and Relative Liver Weights

**Figure 1:**
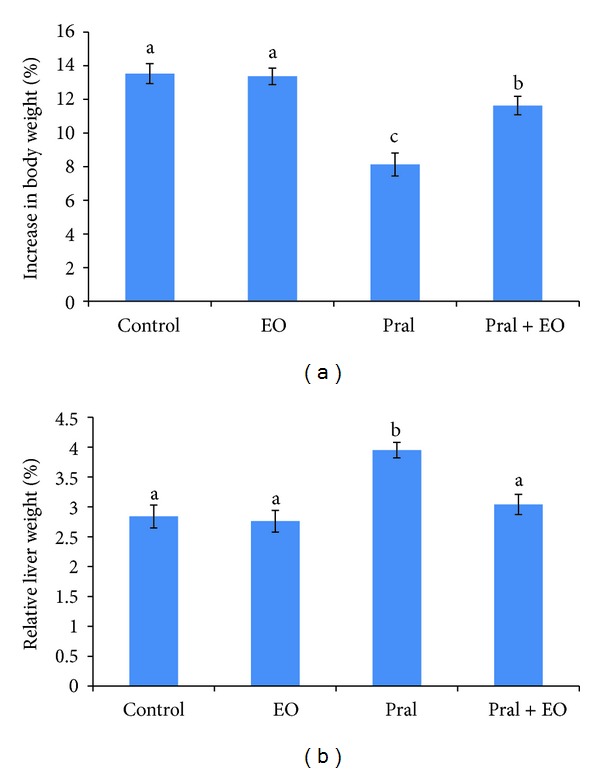
Body (a) and relative liver (b) weights of rats exposed to prallethrin (Pral) and the protective effect of *Origanum majorana* essential oil (EO). Each value is a mean of 7 rats ± SE;  ^a,b,c^ values are not sharing superscripts letters (a, b, c) differ significantly at *P* ≤ 0.05. Increase in body weight (%) = ((final b.wt. − initial b.wt.)/initial b.wt.) × 100. Relative liver weight (%) = (liver weight/body weight) × 100.

Compared to controls, a statistically significant decrease in body weights gain (%) of prallethrin-treated group (8.13% versus 13.53%) and prallethrin + EO-treated group (11.63% versus 13.53%) was recorded (Figure 1). The relative liver weight of prallethrin-treated animals showed a significant increase compared to control (3.95% versus 2.84%). While insignificant changes were observed in relative liver weights of prallethrin + EO and EO-treated groups compared to control (Figure 1).

### 3.3. Hepatic Function Enzymes

**Figure 2:**
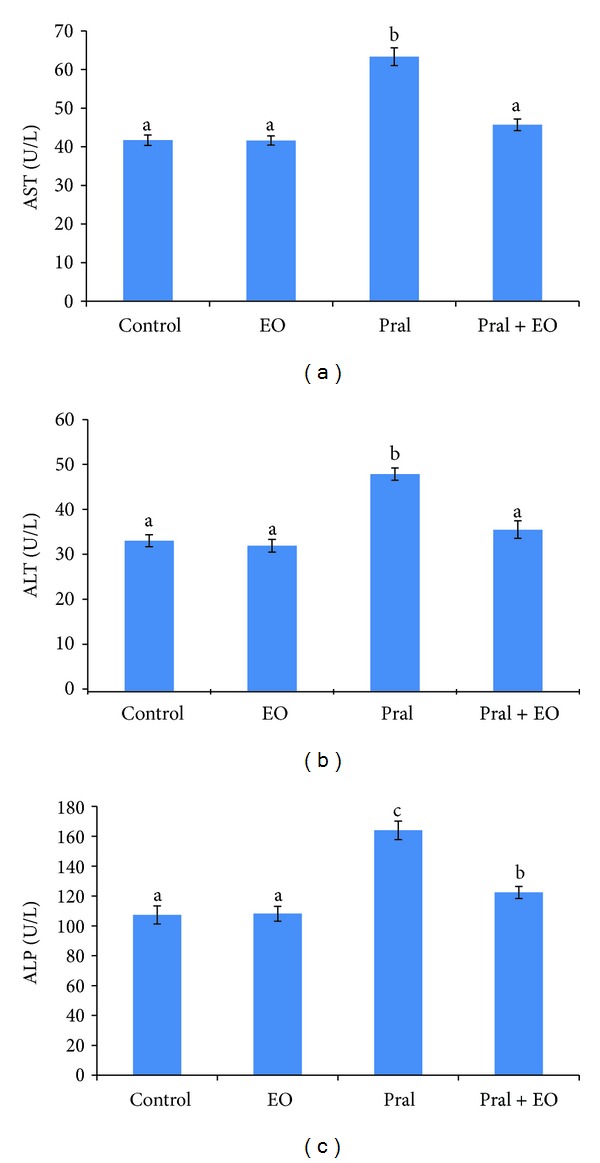
AST (a), ALT (b), and ALP (c) activities in the sera of rats exposed to prallethrin (Pral) and the protective effect of *Origanum majorana* essential oil (EO). Each value is a mean of 7 rats ± SE;  ^a,b,c^ values are not sharing superscripts letters (a, b, c) differ significantly at *P* ≤ 0.05.

As shown in Figure 2, prallethrin-induced hepatotoxicity reflected by elevated serum ALT, AST, and ALP levels (*P* ≤ 0.05). Insignificant changes were observed after EO treatment. Coadministration of EO with prallethrin comparatively and markedly significantly reduced the activities of ALT, AST, and ALP compared with prallethrin group. Compared to controls, AST (45.70 U/L versus 41.72 U/L) and ALT (53.94 U/L versus 33.49 U/L) returned to control values of EO + prallethrin-treated group, while the increase of ALP was significant (122.39 U/L versus 107.34 U/L).

### 3.4. Effect on Lipid Peroxidation

**Figure 3:**
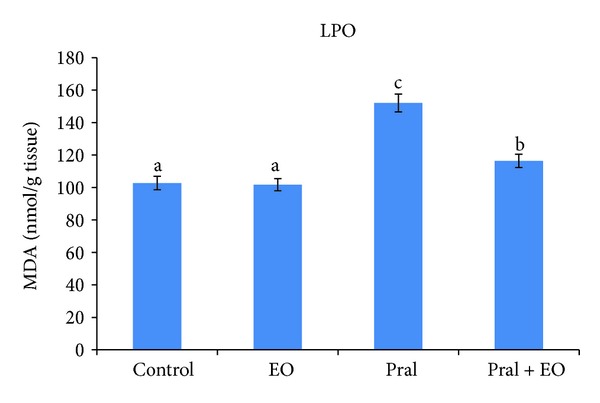
Effect of prallethrin (Pral) and *Origanum majorana* essential oil (EO) coadministered with Pral on the MDA levels in rat liver. Each value is a mean of 7 rats ± SE;  ^a,b,c^ values are not sharing superscripts letters (a, b, c) differ significantly at *P* ≤ 0.05.

Liver MDA level was markedly increased by prallethrin administration as compared to control group. The difference between the two groups was statistically significant (152.07 nmoles of MDA/g tissue versus 102.77 nmoles of MDA/g tissue). EO administered to rats of prallethrin + EO group alleviated lipid peroxidation induced by prallethrin treatment and modulated significantly (116.46 nmoles of MDA/g tissue versus 102.77 nmoles of MDA/g tissue) the levels of MDA in liver compared to control. Results indicated that treatment with EO produced a significant reduction in MDA in prallethrin-treated rats; however EO per se did not alter MDA (Figure 3).

### 3.5. Effect on Antioxidant Enzymes

**Figure 4:**
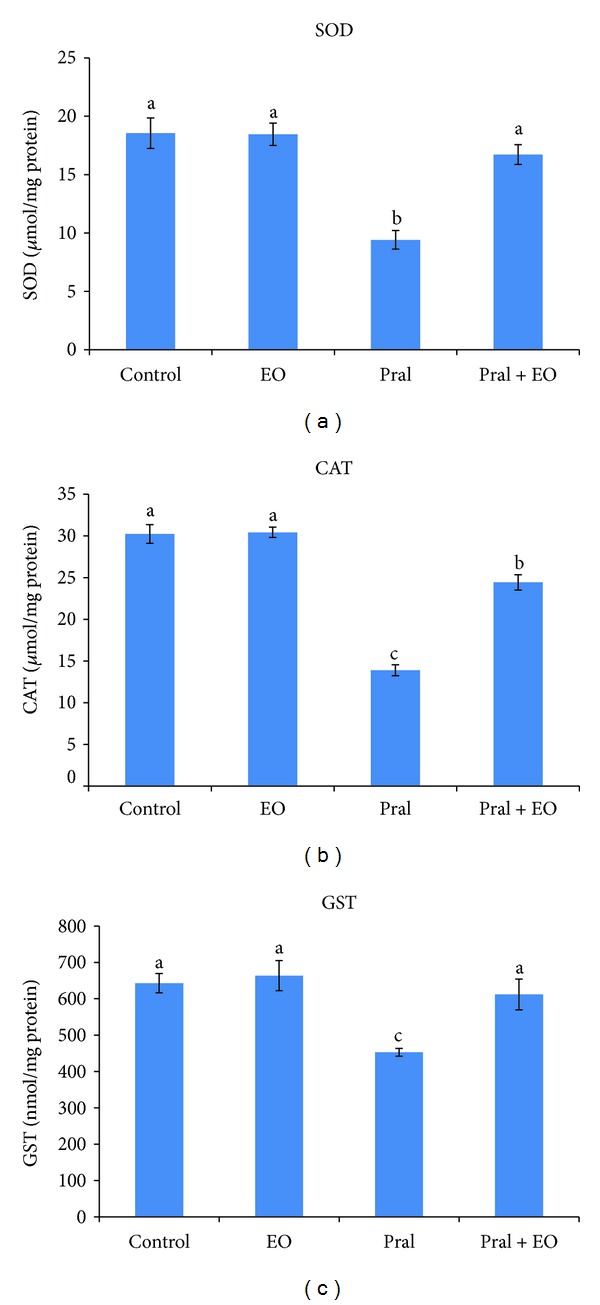
Effect of *Origanum majorana* essential oil (EO) on prallethrin (Pral)-induced alterations in SOD, CAT, and GST activities in liver tissue of control and treated rats. Each value is a mean of 7 rats ± SE;  ^a,b,c^ values are not sharing superscripts letters (a, b, c) differ significantly at *P* ≤ 0.05.

The effects of prallethrin treatment on the activities of SOD, CAT, and GST in liver tissue are shown in Figure 4. Activities of CAT (13.90 *μ*mol/mg protein versus 30.22 *μ*mol/mg protein) and GST (452.64 *μ*mol/mg protein versus 643.01 *μ*mol/mg protein) in liver were significantly decreased compared to control group. EO administrated in prallethrin-treated rats improved significantly the activities of CAT, SOD, and GST in liver compared with control values. The activity of CAT and SOD was returned to control values in prallethrin + EO-treated group, while the decrease of GST was significant compared with untreated group (Figure 4).

### 3.6. Histological Changes

**Figure 5:**
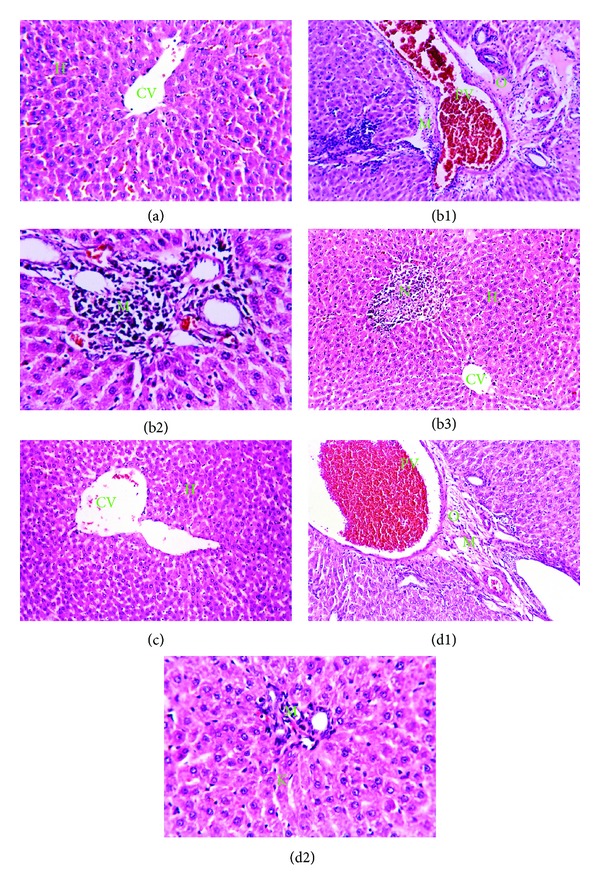
Paraffin sections of liver stained by haematoxylin and eosin (H&E) for histopathological changes. Control group (a) showing the normal histological structure of the central vein (CV) and surrounding hepatocytes (H) (x64). Prallethrin group showing (b1) severe dilatation and congestion of the portal vein (PV) with oedema (O) in portal area (x40), (b2) massive number of inflammatory cells infiltration (M) in the portal area (x80), and (b3) focal necrosis (N) in the hepatic parenchyma (x40). *O. majorana* (EO) group (c) showing intact normal histopathological structure of the central vein (CV) and surrounding hepatocytes (x40). Prallethrin-*O. majorana* (EO) group showing (d1) dilation and congestion in the portal vein (PV), oedema (O), and few inflammatory cells infiltration (M) with dilated bile duct (bd) in portal area (x40) and (d2) diffuse kupffer cells proliferation (K) in between the hepatocytes (x80).

The histopathological changes were graded and summarized in (Table 1). As shown in Figure 5, liver sections stained with H&E showed normal histological structure of the central vein and surrounding hepatocytes in control groups (Figure 5(a)). In prallethrin-treatment group, dilatation and congestion of the portal vein, oedema, infiltration of inflammatory cells, and necrosis were recorded (Figures 5(b1)–5(b3)). Sections of liver from rats, treated with EO alone, showed to be similar to control (Figure 5(c)). Coadministration of EO to prallethrin-treated rats showed dilation, congestion, oedema, few inflammatory cells infiltration, and diffuse kupffer cells and did not reveal any necrosis area (Figures 5(d1) and 5(d2)). However, quantitative assessment of histopathological injury, based on scoring severity of injury in the liver, showed mild to moderate injury after EO coadministration to prallethrin-treated rats (Table 1).

## 4. Discussion

In toxicological studies, body, organ weights, and biochemical parameters are measured to evaluate a broad range of physiological and biochemical functions, affected target organ identification, and tissue injury assessment. In the present study, rats treated with prallethrin at 64.0 mg/kg b.wt. (1/10 LD_50_) daily for 28 days showed no mortality or signs of toxicity throughout the experimental period. Also, food and water consumption were not significantly affected (untabulated data). Our results revealed that treatment of prallethrin caused significant reduction in rat body weight gain while increased relative liver weight compared to control group. In addition, coadministration of EO prevented the toxicity of prallethrin. Increase in liver weight in prallethrin intoxication rat may be due to the increment of biotransformation enzymes [38–40]. Long-term feeding studies with laboratory animals have shown adverse effects of pyrethroid (e.g., cypermethrin); it caused reduced growth rate and increased liver weight in rats [38, 41]. Findings of present study are consistent with previous studies with different pesticides on mammals [42–44].

Liver is a target organ and plays a major role in detoxification and excretion of many endogenous and exogenous compounds. It plays important role in metabolism [45] and biotransformation of toxic compound [46]. Therefore, any type of injury or impairment of its function produces hepatotoxicity and causes health complications. Liver biomarker enzymes, for example, AST, ALT, and ALP, have been commonly associated with liver dysfunction/damage. Hayes et al. [47] reported that one of the indicators for liver damage and function is increase in the activities of transaminases (AST and ALT) in the serum. They play a role in amino acids catabolism and biosynthesis. ALP mainly reaches the liver from bone, excreted into the bile; therefore its elevation in serum can be associated with hepatobiliary disease [48]. The present study revealed that prallethrin-induction in rats remarkably increased the level of ALT, AST, and ALP. This increase may be indicative of initial cell injury occurring in advance of gross hepatic pathology. It causes hepatocyte injuries and altered membrane integrity and as a result enzymes in hepatocytes leak out [49]. The activities of transaminases and ALP were increased in rat after exposure to prallethrin [50], permethrin [51], fluvalinate [52], cypermethrin and deltamethrin [53], and fenvalerate in buffalo calves [54]. However, coadministration of EO to prallethrin intoxicated rats decrease ALT, AST, and ALP activity to within normal levels. These results indicated the ability of EO to protect against prallethrin-induced hepatocyte injury, which is in agreement with a previous study [55] that reported the protective consequence of polyphenolic compounds against xenobiotic-induced liver injury.

Reactive oxygen species (ROS) are causally related to oxidative stress. Many studies have demonstrated that overproduction of ROS can further aggravate oxidative stress and have implicated ROS in a number of disease processes, including heart disease [56], diabetes [57], liver injury [19, 20, 43, 58, 59], cancer [60], and aging [61]. Maintaining the balance between ROS and antioxidant enzymes, such as superoxide dismutase (SOD), catalase (CAT), and glutathione-s-transferase (GST), is, therefore, crucial and could be an important mechanism for preventing damage by oxidative stress. This balance has been suggested to have an important role in preventing pesticides toxicity [19, 20, 43, 58, 59].

In fact, liver was the major site of pyrethroid metabolism which accumulated a great concentration of its metabolites [62, 63]. Their toxic effects occurred probably through generation of reactive oxygen species causing damage to various membranous components of the cell. Our results revealed that prallethrin caused a statistically significant decrease in the activity of SOD, CAT, and GST in liver of rats. SOD catalyses the dismutation of superoxide anion (O_2_^∙−^) to H_2_O_2_ and O_2_. Because H_2_O_2_ is still harmful to cells, CAT catalyses the decomposition of H_2_O_2_ to water. GST is a detoxifying enzyme that catalyzes the conjugation of a variety of electrophilic substrates to the thiol group of GSH, producing less toxic forms [64]. Thus, the coordinate actions of various cellular antioxidants in mammalian cells are critical for effectively detoxifying free radicals. Therefore, any impairment in this pathway will affect the activities of other enzymes in the cascade [65, 66]. However, reduction in the activity of SOD will result in an increased level of O_2_^∙−^, while a decrease in the activity of CAT will lead to accumulation of H_2_O_2_ in the cell, which leads to peroxidation of membrane lipids via Fenton-type reaction. TBARS, the final metabolites of peroxidized polyunsaturated fatty acids, are considered as a late biomarker of oxidative stress [67] and are a good indicator of the degree of lipid peroxidation [16]. In the present study, we observed significant increase (*P* ≤ 0.05) in the levels of TBARS in liver of prallethrin-treated rat. Our results revealed that prallethrin exposure induces oxidative stress in the liver of rat as indicated by decreased SOD, CAT, and GST activity and elevated TBARS concentrations, which would further induce lipid peroxidation, initiate free radicals damage to hepatocellular membrane, and lead to liver injury. The possible explanation for this effect could be that the increase in the formation of liver lipid peroxidation in prallethrin-intoxicated animals acted as a signal to maintain lower levels of antioxidant enzymes (SOD, CAT, and GST) in order to enhance the triggering of the detoxification process for the pyrethroid. So, the decrease in the activity of SOD, CAT, and GST in prallethrin-intoxicated animals indicates insufficient detoxification of prallethrin in rats. The decline of antioxidant enzymes activity, in our study, supported earlier findings [68, 69] which demonstrated that exposure of rats to pyrethroids decreased antioxidant enzymes activity. The increment in lipid peroxidation, as assessed by the elevated levels of TBARS following insecticides administration, has been well documented [16, 19, 29, 59, 70, 71]. Cypermethrin exposure to rats resulted in free radical-mediated tissue damage as indicated by elevated cerebral and hepatic lipid peroxidation [62]. Cypermethrin and fenvalerate increased the oxidative stress and LPO in liver, kidneys, and heart tissues of rats [72]. Our results corroborated previous reports [73–75]who have demonstrated that pyrethroids exposure like fenvalerate and deltamethrin altered antioxidant defense mechanisms and enhanced lipid peroxidation in rat liver. Therefore, oxidative stress and LPO has been implicated in the toxicology of pyrethroids [70, 71]. In fact, LPO alters the physiological functions of cell membranes and plays an important role in cellular membrane damage. It has been shown to perturb the bilayer structure and modify membrane properties such as membrane fluidity, permeability to different substances, and bilayer thickness.

The elevation of AST, ALT, and ALP enzymes activity in this study suggests probable liver tissue damage due to prallethrin as evidenced by the histopathological lesions like dilatation, congestion, oedema, inflammatory cells, and necrosis. Regardless of the causing agent of the hepatic lesion, the liver will apparently react in five ways: (1) necrosis, (2) degeneration, (3) inflammation, (4) regeneration, and (5) fibrosis. Necrosis may follow practically any lesion whose changes are significant, taking a toll on hepatocytes. However, before it becomes characteristically necrotic, hepatocytes may become swollen and edematous, with irregularly compact cytoplasm and great clear spaces. The decrease of CAT, SOD, and GST activities and increased TBARS level suggest that prallethrin causes hepatic damage and pathogenesis may be through the generation of free radicals and oxidative damage which certainly play a vital role in the pathogenesis of liver injury. The present study has demonstrated that the EO exerts a hepatoprotective effect against prallethrin-induced hepatotoxicity in rat. Increased levels of antioxidant enzymes and a reduction in the amount of lipid peroxides are likely to be the major mechanisms by which EO prevents development of the liver damage induced by prallethrin. Supporting this hypothesis, we observed significant increase in SOD, CAT, and GST activity and decrease in the levels of TBARS in liver of prallethrin-treated rat by the administration of EO. This might be due to hydroxyl radicals scavenging activities of EO. The EO of *O. majorana* shows potent antioxidant activity and many antioxidant components are found in EO [76–78]. The high potential of phenolics components to scavenger radicals might be explained by their ability to donate a hydrogen atom from their phenolic hydroxyl groups [27]. It contains phenolic terpenoids (thymol, carvacrol), flavonoids (diosmetin, luteolin, and apigenin), tannins, hydroquinone, phenolic glycosides (arbutin, methyl arbutin, vitexin, and orientinthymonin), triacontane, sitosterol, oleanolic acid and cis-sabinene hydrate [79–82]. It has been reported that the antioxidant activity of EOs could not be attributed to the major compounds, and minor compounds might play a significant role in the antioxidant activity, and synergistic effects were reported [77]. In the present study, the most prominent components of *O. majorana L.* EO were 4-terpineol (29.97%), *γ*-terpinene (15.40%), trans-sabinene hydrate (10.93), *α*-terpinene (6.86%), and 3-cycolohexene-1-1 methanal,a,a4-trimethyl-,(S)-(CAS) (6.54%) [27]. Therefore, the possible mechanisms of *O. majorana* EO hepatoprotective could arise from the free radical scavenging effect, preventing lipid peroxidation and improvement of the antioxidant/detoxification system in liver. Furthermore, the free radical scavenger effect of *O. majorana* EO has been reported by many authors [77, 83–86]. Several studies have indicated that treatment with antioxidants can ameliorate the toxicity of pyrethroids [71, 87].

## 5. Conclusion 

In view of the data of the present study, it can deduce that prallethrin caused oxidative damage and liver injury in male rats. These results could be useful for increasing information on the potential toxicity of this pyrethroid. The coadministration of *O. majorana* EO attenuated the toxic effect of prallethrin. These results demonstrate that administration of EO appeared to be a promising agent for protection against prallethrin-induced oxidative damage and hepatotoxicity. Therefore, administration of *O. majorana* may be useful, easy, and economical to protect humans exposed to pyrethroids against their toxic effects.

## Figures and Tables

**Table 1:** Histopathological changes in the liver of male rats exposed to prallethrin (Pral) and the protective effect of *Origanum majorana* essential oil (EO), based on scoring severity of injury.

Observation	Control	EO	Pral	Pral + EO
Inflammatory cells in the portal area	−	−	+++	+
Focal necrosis in the hepatic parenchyma	−	−	++	−
Diffuse kupffer proliferation	−	−	−	++

Normal (−), minimal (+), mild (++), moderate (+++).
